# The Relationship Between Sjogren’s Syndrome and Sleep Disturbance: A Case Report

**DOI:** 10.7759/cureus.30321

**Published:** 2022-10-15

**Authors:** Baiazit Tcholakov, Hodan Qasim

**Affiliations:** 1 Family Medicine, Poznan University of Medical Sciences, Poznan, POL; 2 Internal Medicine, Alfaisal University, Riyadh, SAU

**Keywords:** autoimmune, sleep, abnormal sleep, insomnia, primary sjogren’s syndrome

## Abstract

Sjogren’s syndrome is an autoimmune disorder characterized by the infiltration and disruption of exocrine glands by the host’s immune cells. It is the third most common autoimmune syndrome after systemic lupus erythematosus (SLE) and rheumatoid arthritis (RA). This syndrome most commonly affects females. This disorder most typically presents as sicca symptoms, though plenty of other symptoms can exist. One of these other symptoms is the disturbance of sleep. The insomnia component of Sjogren’s syndrome is the focus of this case report.

## Introduction

Sjogren’s syndrome is typically diagnosed in the fifth decade of life with an incidence of 6.9 and a prevalence of 60.8 per 100,000 [1]. The etiology is unknown but Sjogren’s affects females disproportionately more since there are nine females for every male. The pathogenesis in Sjogren’s syndrome is related to epithelial cells where these cells both trigger and target the immune reaction [1].

It is found that the most important risk factor is being female. The X chromosome is heavily suggested to be the major source of possible genes that cause Sjogren’s. The major histocompatibility complex (MHC) genes most associated with primary Sjogren’s syndrome (pSS) are the *DR2* and *DR3* alleles at the DRB1 locus. The genes with significant connection are the *HLA-DQB1* and the *HLA-DQA1*. In a couple of European cohorts, the MHC class I polypeptide-related sequence A (MICA) gene had a relationship with pSS. In Han Chinese patients, the genes most associated were the *HLA-DRB1/HLA-DQA1* and *HLA-DPB1*. There are other genes that have an association with Sjogren’s syndrome. The genes interferon (IFN) regulatory factor 5 (IRF5), signal transducer and activator of transcription 4 (STAT4), interleukin-12 subunit alpha (IL12A), and 2′-5′ oligoadenylate synthetase 1 (OAS1) are all related to type 1 IFN signaling. As for the epigenetics, in the peripheral blood cells, the hypomethylation of sites in IFN-induced genes tended to have an association with Sjogren’s. Specific genes include *MX1*, *IFI44L*, *PARP9* and *IFITM1*. There is more hypomethylation in people with positive anti-Ro/Sjogren’s syndrome A (SSA) and/or anti-La/Sjogren’s syndrome B (SSB) antibodies. Such epigenetics is also present in salivary gland cells, the hypomethylation of sites in IFN-induced genes, but affecting OAS2 [2].

Clinical manifestations typically include glandular involvement of the eyes and mouth. Ocular inflammation of eye surfaces presents clinically with eye erythema, itching, and photosensitivity and in the long term can lead to corneal ulceration. Oral (mouth) dryness can present with glandular swelling, and symptoms can include dysphagia, pain, dental caries, and eventually infection and inflammation. Patients with Sjogren’s report fatigue, as well as anxiety, depression, and sleep disorder. Additional clinical manifestations can include myalgia, arthralgia, xerosis, Raynaud phenomenon, pulmonary involvement, cardiac involvement, neural involvement, renal involvement, and GI involvement. Finally, there is a chance that the condition can lead to lymphoma, typically B-cell non-Hodgkin lymphomas and mucosa-associated lymphoid tissue lymphomas (MALTomas) [1].

The diagnosis of Sjogren’s syndrome is determined by the American College of Rheumatology/European Alliance of Associations for Rheumatology (ACR/EULAR) criteria method. Methods for assisting in diagnosing Sjogren’s include an antibody assay, Schirmer’s test, ocular staining, and sialometry. A salivary gland biopsy is diagnostic [1,3]. To test for sicca symptoms, Schirmer’s test and Saxon’s test are possible methods and are easy to perform. However, they don’t always correlate with patient symptoms. Topical vital stains can visualize lesions due to the sicca symptoms. The measurement of salivation is the gold standard; however, the procedure is rather time-consuming to be done in a clinic. Ultrasonography (USG), while not part of the classification criteria, can be used. Testing for antinuclear antibody (ANA) alone is not enough; the presence of anti-Ro/SSA or anti-La/SSB antibodies will help in diagnosing pSS. However, patients with other connective tissue disorders can have positive anti-Ro/SSA antibodies. Thus, other clinical clues would be needed for the diagnosis. Biomarkers can monitor disease progression, one such marker being the Siglec-1; it correlates with disease activity. Histopathology is also useful. Finding focal periductal localized lymphocytic infiltrates in these glands points toward pSS. Most of the infiltration are CD4+ T cells. A minimum number of 50 monocytic cells/4 mm² was defined as a focus score (FS) of 1. At least FS of 1 would correlate with pSS [3].

Targeted treatment is still not available. Current treatment is on an individual basis. With secondary conditions, treatment is primarily around the underlying condition. Disease-modifying therapy would be used for patients with extraglandular manifestations. The measures of these manifestations are done by the EULAR Sjogren’s Syndrome Disease Activity Index (ESSDAI). For the majority of patients, therapy is based around the management of the fatigue and sicca symptoms. Patient education is a part of symptom management, which focused on environmental factors and habits. Medications used for the sicca symptoms include tear substitutes and anti-inflammatory eye drops. Xerostomia is complicated to treat due to the complex physiology of saliva secretions. The symptom can be alleviated. Dental work is difficult on these people, though implants make good outcomes. Disease-modifying antirheumatic drugs (DMARDs) have questionable effectiveness, though more studies are needed. In regard to immunosuppression, it is ultimately determined by organ involvement. High dose of methylprednisolone and cyclophosphamide is given to patients with severe organ involvement. Cryoglobulinemia, rituximab, or plasmapheresis is given to patients with vasculitis. For pregnant females, hydroxychloroquine is recommended if she has a risk of congenital heart block. New treatments are currently undergoing evaluation, such as the modulation of B-cell hyperactivity, antagonizing T-cell co-stimulation, or effector cytokines [3].

There is no cure for Sjogren’s syndrome; treatment is based around relieving symptoms. Managing the sicca symptoms is imperative in increasing the quality of life (QoL) for these patients, with the management of ocular dryness having greater impact. Extraglandular symptoms are managed empirically. Sleep disturbance is not classically associated with Sjogren’s, but it is possible [1]. In our present case, the sleep disorder is the point of interest.

## Case presentation

The patient is a 64-year-old male with a past medical history of lumbar pain due to trauma. This was his first visit to this clinic. The patient came presenting with depression and anxiety for several years, which apparently worsened in the past two months. The patient was on Norco (hydrocodone-acetaminophen) for the pain for five years; however, his previous physician can no longer prescribe him the medication. The patient reports that his increased pain is contributing to the increased depression recently. The patient was found to have multilevel degenerative disc disease and facet osteoarthritis, contributing to his back pain. The patient also reports tinnitus, as well as insomnia. He had difficulty falling asleep every night and as a result has decreased energy and concentration. The patient also brought up that he had dry mouth and dry eyes, prompting the physician to draw blood for an ANA screen. The patient was started on escitalopram for anxiety. The patient was also started on Norco for the pain.

ANA reflex came back positive with ribonucleoprotein (RNP)/Smith (Sm) and SSB above normal level. Single-stranded DNA (ssDNA) was also above the normal level. This is highly diagnostic for Sjogren’s syndrome. The patient came in for a follow-up three months later. The patient was diagnosed with Sjogren’s syndrome based on the lab results. Also, the patient reports averaging four hours of sleep every night and needing to use lorazepam twice daily to fall asleep. The patient says that the symptoms of Sjogren’s syndrome would cause him to wake up at night, contributing to his limited sleep time. The patient also notes that his back pain and tinnitus might be contributing to his sleep disturbances. The escitalopram dose was increased, and the patient was started on prednisone, carboxymethylcellulose sodium, and zolpidem.

## Discussion

Prior studies found connections between sleep loss and the presence of Sjogren’s. One of these studies was a focus group study by Hackett et al. [4]. Sixty-two possible participants were contacted for participation, and 13 were able to show up. A patient in the focus group study indicated that achieving sleep was effortless. After the disease onset, however, she experienced a decrease in sleep quality and an increase in difficulty in falling asleep. Another patient would struggle to fall asleep, often lying awake at night. Other patients would awake at night. One often is awake for hours before falling back to sleep, the effect being an unfulfilling sleep [4].

A systematic review of the literature by Hackett et al. was performed to investigate the connection between sleep disturbances and Sjogren’s syndrome [5]. In four of the studies, the patient’s sleep disturbance was measured by questionnaires or sleep diaries. The outcome is that patients with primary Sjogren’s syndrome score significantly worse than controls [5]. A cross-sectional study from Brazil provides important information on the relationship between sleep and Sjogren’s (Dardin et al.) [6]. The purpose of the three-year-long clinical trial was to assess the link between Sjogren’s syndrome and the quality of life. This was tracked by several questionnaires and scales, including the visual analog scale for pain (VAS Pain), EULAR Sjogren’s Syndrome Patient Reported Index (ESSPRI), EULAR Sjogren’s Syndrome Disease Activity Index (ESSDAI), Profile of Fatigue and Discomfort-Sicca Symptoms Inventory Short Form (PROFAD-SSI-SF), visual analog scale for fatigue (VAS Fatigue), Pittsburgh Sleep Quality Index (PSQI), and Medical Outcomes Survey Short Form-36 (SF-36) [6].

The Pittsburgh Sleep Quality Index (PSQI) measured sleep quality. Only a quarter of the patients had good sleep quality [6]. The other three-quarters reported poor sleep quality. The clinical trial found a significant positive correlation between sleep disturbance and disease activity. There was also a correlation between sleep quality and pain, fatigue, and symptoms. They made mention of additional studies that saw the same relationship between sleep quality and Sjogren’s syndrome. They found that people with Sjogren’s had sleep disorders [6]. A study exploring the link between sleep and Sjogren’s also evaluated the presence of anxiety and depression in these patients. In this cohort study, 86% of patients had an abnormal memory because of Sjogren’s. Thirty-two percent of people with Sjogren’s syndrome had fatigue. Median pain score was 6 out of 10. Forty percent of patients had depression. Forty-eight percent had a mild to severe anxiety trait. In terms of sleep, 71% of these patients had insomnia. Also, 55% had daytime sleepiness [7]. Generally, there are more sleep disturbances in people with Sjogren’s than people without Sjogren’s. An additional example is a patient in our case, who reported difficulty falling asleep every night. We can take a closer look at how Sjogren’s affects aspects of sleep.

One such aspect is time in bed. Time in bed was looked into by two studies from the systematic review with differing methodologies [5]. One study had the usage of polysomnography, while the other utilized a sleep questionnaire. While the polysomnography study saw no significant difference between pSS and controls. The outcome of the polysomnography can be up to question due to the small sample size, and the nature of the polysomnography protocol may have influenced the results of the study [5]. Another aspect is the duration of sleep, distinct from time in bed. Five of the studies from the systematic review measured sleep duration, but the outcome was rather conflicted [5]. Three small studies found no significant difference between the total sleep time of people with Sjogren’s syndrome and controls. However, one sleep diary study found that there was a significant difference between the sleep duration of people with Sjogren’s syndrome and healthy controls. They found that people with Sjogren’s had 40 minutes to one hour and 45 minutes less sleep via sleep diary. The polysomnography study showed a decrease of one to two hours of sleep [5]. Three studies measured the amount of sleep in each stage. Two of the studies noted that people with Sjogren’s syndrome spent more time in stage 1 sleep than controls [5]. Our patient had expressed that he had, on average, four hours of sleep a night.

A ratio of sleep duration to time in bed can net sleep efficiency. Two studies found that a percent of time spent in bed asleep was reduced for people with Sjogren’s syndrome [5]. There was a study exploring sleep characteristics of people with Sjogren’s syndrome (Lewis et al.) [8]. Phase two involved examining clinical data from a cohort of patients. The patients completed questionnaires and sleep diaries. Phase two showed that people with Sjogren’s syndrome tended to have problematic sleep. These patients would have more nighttime awakenings and have lower sleep efficiency, meaning they spent more time in bed without sleeping. The patients with Sjogren’s had an overall lower quality of sleep [8]. All of the studies in the systematic review noted that there is an increased number of nighttime awakenings in Sjogren’s patients. Another aspect of sleep that may be affected by Sjogren’s is sleep latency or the time it takes for an individual to fall asleep. The usage of polysomnography found that there was a difference in sleep onset latency between people with the syndrome and controls, the difference being seven minutes [5]. Actigraphy measured a mean of 26.2 minutes for sleep latency and a mean of 48.2 minutes for nighttime awakenings. The sleep efficiency was reported to be 89.7%, and on average, the patients were achieving about six and a half hours of sleep. As compared to the healthy controls, people with Sjogren’s had double the sleep latency [6].

The lack of sleep during the night can lead to daytime sleepiness. One patient in the focus study was reported to regularly fall asleep during the day. Some other patients had unplanned naps throughout the day. Another patient, in particular, had fallen asleep during one of the focus group discussions [4]. Within the systematic review, four of the studies found that patients with primary Sjogren’s syndrome experienced increased daytime sleepiness [5]. During phase one of the Lewis et al. study, they explored sleep disturbances through data from the UK Primary Sjogren’s Syndrome Registry. This phase was to explore daytime hypersomnolence. The results of phase one was a significant increase in daytime hypersomnolence for patients with Sjogren’s syndrome. Also, there was the presence of a correlation between hypersomnolence and fatigue for these patients [8]. Our patient with Sjogren’s had mentioned having decreased energy and difficulty concentrating.

Why is there a link between Sjogren’s syndrome and sleep disturbance? A highly possible source of the sleep disturbance is the symptoms of Sjogren’s causing enough discomfort to affect the patient’s sleep. From the Hackett et al. focus study, the relationship between Sjogren’s and sleep interference is explained as being caused by the symptoms of the condition. Some patients believed that the dryness caused discomfort that would awaken the patients. Another symptom causing sleep disturbance was leg discomfort [4]. The Hackett et al. systematic review also touched on this [5]. A number of Sjogren’s patients report that the sicca symptoms can cause sleep difficulty. Patients with primary Sjogren’s syndrome experienced more nocturnal sweating than rheumatoid arthritis (RA) and the controls. They also had more palpitations [5]. Dardin et al. found that, unsurprisingly, patients with Sjogren’s syndrome generally experienced more pain than those without the condition [6]. The patients also experienced both physical fatigue and mental fatigue. The patients had ranked their quality of life according to the Medical Outcomes Survey Short Form-36 (SF-36), with the highest score being 61.5 (for functional capacity) and the lowest being 34.5 (for physical aspects) [6]. The study reiterated that there was a prevalence of fatigue in Sjogren’s patients. Quality of life was worse with people with Sjogren’s syndrome, and that was reflected by the healthcare cost. Fatigue and social and psychological factors are what contributed to the increased cost. However, the glandular manifestations were a nonfactor for the healthcare cost [6].

Generally, the quality of life of Sjogren’s patients was lower [6,7]. While patients with systemic involvement of Sjogren’s generally also had pathologic cognition, there was no association with increased depression, anxiety, sleep disorders, fatigue, pain, or worse quality of life [7]. People with Sjogren’s with a low quality of life also generally did not have higher depression, higher anxiety, more sleep disturbance, or fatigue [7]. Generally, younger patients with less systemic disease had more fatigue; they also had increased pain, worse quality of life, and daytime sleepiness [7]. ESSDAI level had no correlation to cognitive disorders, sleep disorders, fatigue, or quality of life. Cognitive issues did not show as pathologic brain MRIs [7]. There was a cross-sectional study that compared people with rheumatoid arthritis and Sjogren’s syndrome to healthy controls [9]. Patients with Sjogren’s syndrome had significantly lower quality of life scores than healthy controls, but there were no significant differences in the physical component of the scoring of QoL [9]. People with RA had lower physical component scores [9]. Patients with Sjogren’s syndrome also had a significantly higher amount of fatigue than controls, while people with RA did not differ in terms of fatigue [9]. People with Sjogren’s syndrome had poor sleep compared to control, and they were not as refreshed on awakening [9]. There was no significant difference between the RA and control group in terms of sleep quality [9]. However, wrist actigraphy was used as a method of measuring sleep quality [9]. This method did not find any significant difference in sleep quality, sleep efficiency, or sleep time between the RA, pSS, and control groups. It also showed that the RA group had a slight increase in awakenings [9]. Our patient reported that the lack of sleep was due to many factors. The symptoms of Sjogren’s syndrome was reported to be a cause of the sleep disturbance. Some other symptoms causing the shortage of sleep are tinnitus and back pain that the patient also has.

The Hackett et al. focus group study pondered probable solutions to this side effect. Patients have made attempts to relieve their sleep issues [4]. Strategies such as power napping and meditation had varying levels of success. Sleeping pills, however, were not well received by the patients. Part of the study was to measure sleep outcomes by the usage of components of cognitive behavioral therapy for insomnia (CBT-I). The result shows the benefits of these components on sleep difficulty. For one, it increased patient awareness of their sleep issues and their connection with their condition. It also gave them the motivation to attempt to resolve their sleep issues by the application of the techniques from CBT-I. However, patients typically expect immediate results from these techniques [4].

## Conclusions

With the sum of these prior studies, there may be a connection between sleep disturbance and difficulty and Sjogren’s syndrome. People suffering from the condition tend to have a longer sleep latency. They also tend to have frequent nighttime awakenings and struggle to fall back to sleep. This would decrease sleep efficiency as they would remain in bed without sleeping, reducing sleep time. During the day, they would experience daytime hypersomnolence and would typically nod off into sleep. The cause of the sleep disturbance in Sjogren’s is likely to be the pain and sicca symptoms associated with the disease. As related to this patient, his sleep difficulty is most likely caused by the condition.
